# The solution plasma process for heteroatom-carbon nanosheets: the role of precursors

**DOI:** 10.1038/s41598-017-04190-x

**Published:** 2017-06-19

**Authors:** Koangyong Hyun, Nagahiro Saito

**Affiliations:** 1 Faculty of Engineering, Shinshu University, Nagano, 380-8553 Japan; 2 Graduate School of Engineering, Nagoya University, Nagoya, 464-8603 Japan

## Abstract

The solution plasma process (SPP), known as non-equilibrium cold plasma at atmospheric pressure and room temperature, was used to investigate the synthesis of nitrogen-carbon nasnosheets (NCNs). To verify the effect of elementary composition and structure of *N*-methyl-2-pyrrolidone (NMP), various precursors were used in the SPP to synthesize NCNs via the bottom-up synthesis method for the first time. The NCNs were analyzed by transmission electron microscopy, Raman spectroscopy, and X-ray photoelectron spectroscopy. Among the various precursors, SPP of 2-pyrrolidone was demonstrated to facilitate the formation of highly ordered NCNs. On the other hand, the SPP with cyclopentanone, cyclohexanone and pyrrole did not lead to the formation of carbon nanosheets. The results of this study would uncover new parameter fields for the growth of heteroatom-carbon nanosheets using this synthesis system. In addition, the study is expected to contribute toward research in improving the large-area growth and quality of two-dimensional nanostructures, such as heteroatom-carbon nanosheets or graphene, for various applications in other synthesis methods.

## Introduction

Among various carbon nanomaterials, two-dimensional (2D) carbon allotropes such as carbon nanosheets (CNs), which are composed of few- to multi-layer graphene sheets^1, 2^, have emerged as a promising material for the development of lithium-ion batteries^3^, supercapacitors^4^, organic solar cells^5^, sensitive gas-detection materials^6^, field emission materials^7^, carbon dioxide adsorbents^8^, and fuel cells^9^.

The introduction of heteroatoms (e.g., nitrogen or boron) into CNs, which do not exhibit a band gap by themselves, has been important for tailoring their electronic properties and chemical reactivity by opening the band gap and modulating conducting types^10–14^. In particular, nitrogen has attracted much attention as the most common dopant element because it is similar in size to carbon and contains five valence bonds with carbon atoms^15–17^. Therefore, it can potentially be used for various applications^18–20^.

Recently, the solution plasma process (SPP), known as non-equilibrium cold plasma at atmospheric pressure and room temperature^21^, was used to synthesize CNs via the bottom-up synthesis method for the first time^22^. Nitrogen-carbon nasnosheets (NCNs), composed of multi-layer graphene (MLG) with turbostratic stacking, were synthesized by the SPP at a high-repetition frequency with *N*-methyl-2-pyrrolidone (NMP). The findings demonstrated the advantages of the SPP, such as a short synthesis time, simple experimental apparatus, no impurity issues, and the ability to operate at room temperature, when compared with nanosheets prepared by conventional methods^22, 23^. Before the synthesis of NCNs, various organic precursor solutions have been used in the SPP to produce carbon nanomaterials for use in carbon nanomaterial-based electrocatalysts such as fuel cells and lithium-air batteries^24–27^. Although these syntheses were under the same or similar synthesis conditions as NCNs, they lead only to one type of carbon nanomaterial structure such as a carbon nanoball. The formation mechanism of nanosheets only from the SPP of NMP remains unclear.

In this study, various precursors that have a similar structure to NMP, such as 2-pyrrolidone, 1-methylpyrrolidine, pyrrolidine, pyrrole, cyclopentanone, and cyclohexanone, were used to investigate and reveal the effects of the NMP precursor and its elementary composition. They were analyzed by transmission electron microscopy (TEM), Raman spectroscopy, and X-ray photoelectron spectroscopy (XPS). The results demonstrated the most distinct differences in the carbon structure based on the presence of oxygen or nitrogen as well as the structural differences between each precursor.

The results of this study would uncover new parameter fields for the growth of heteroatom-CNs using this synthesis system. In addition, this study is expected to contribute toward improving the quality and the large-area growth of 2D nanostructures, such as nanosheets or graphene, in other synthesis methods.

## Results

**Figure 1:**
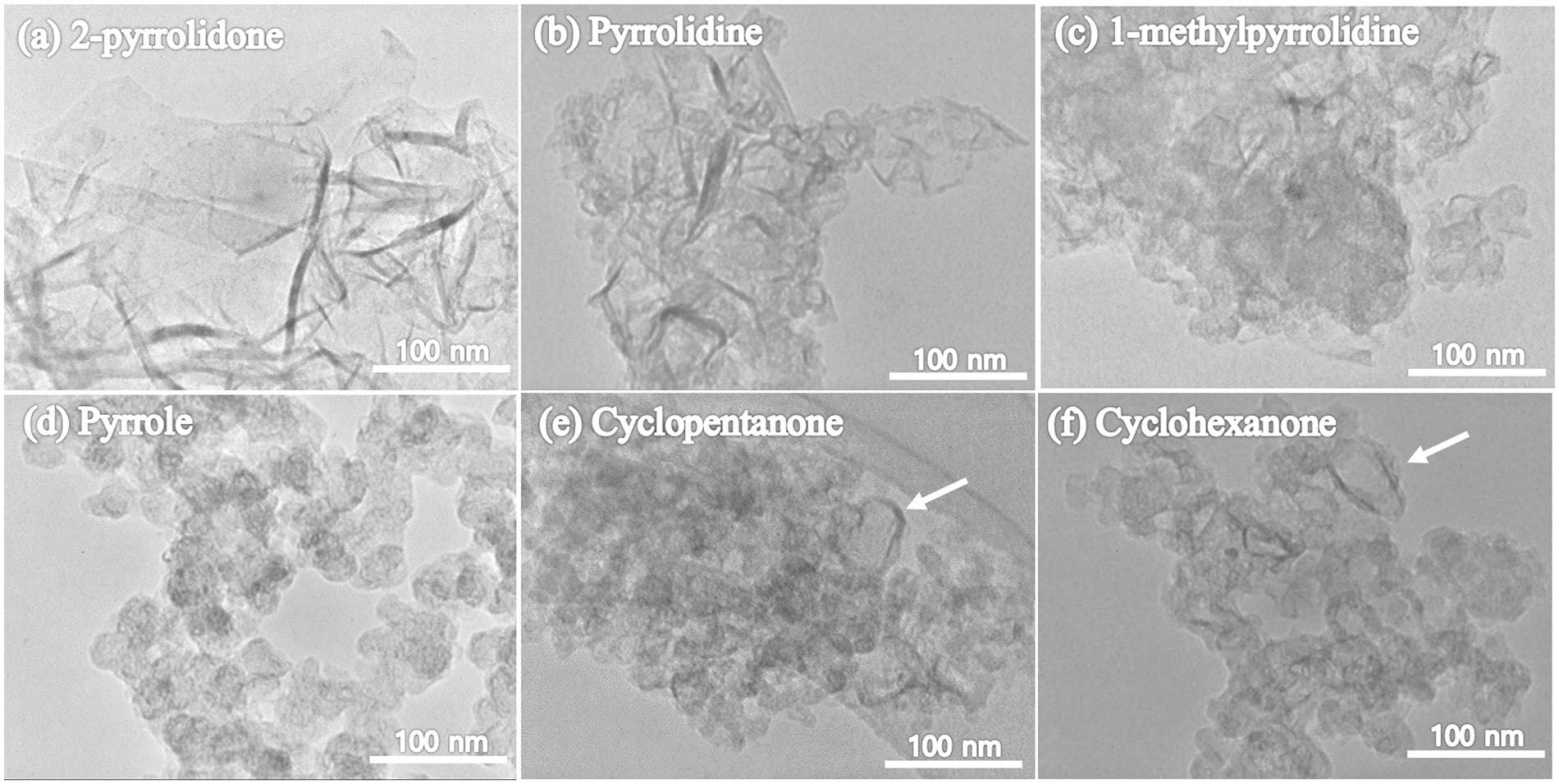
TEM images of the carbon nanomaterials obtained from different precursors: (**a**) 2-pyrrolidone, (**b**) pyrrolidine, (**c**) 1-methylpyrrolidine, (**d**) pyrrole, (**e**) cyclopentanone, and (**f**) cyclohexanone.

Typical TEM images were used to confirm the morphology of the carbon nanomaterials synthesized from 2-pyrrolidone, pyrrolidine, 1-methylpyrrolidine, pyrrole, cyclopentanone, and cyclohexanone by the SPP, as shown in Fig. 1. Interestingly, the SPP of 2-pyrrolidone resulted in the formation of homogeneous NCNs as shown in Fig. 1(a). The material consisted of planar thin and crumpled nanosheets on a carbon grid. No other carbon nanostructures were found in this material. The size of the NCNs prepared from 2-pyrrolidone ranged from hundreds of nanometers to several micrometers, as approximated from scanning electron microscopy images (see Fig. S1). The structures of NCNs in the carbon nanomaterials prepared from pyrrolidine and 1-methylpyrrolidine were assessed as shown in Fig. 1(b) and (c), respectively. The materials were found to consist of randomly aggregated, dense, and thick nanosheets with irregular shapes. The carbon nanomaterials prepared from pyrrole exhibited a substantially different shape by forming a three-dimensional structure of carbon nanoballs as shown in Fig. 1(d). Figure 1(e) and (f), corresponding to the TEM images of the carbon nanomaterials prepared from cyclopentanone and cyclohexanone, respectively, also presented an irregular shape. Partial domains of CNs (marked with arrows) were also found. These results indicated that the morphologies of the carbon nanomaterials prepared by the SPP were strongly dependent on the different precursors.

**Figure 2:**
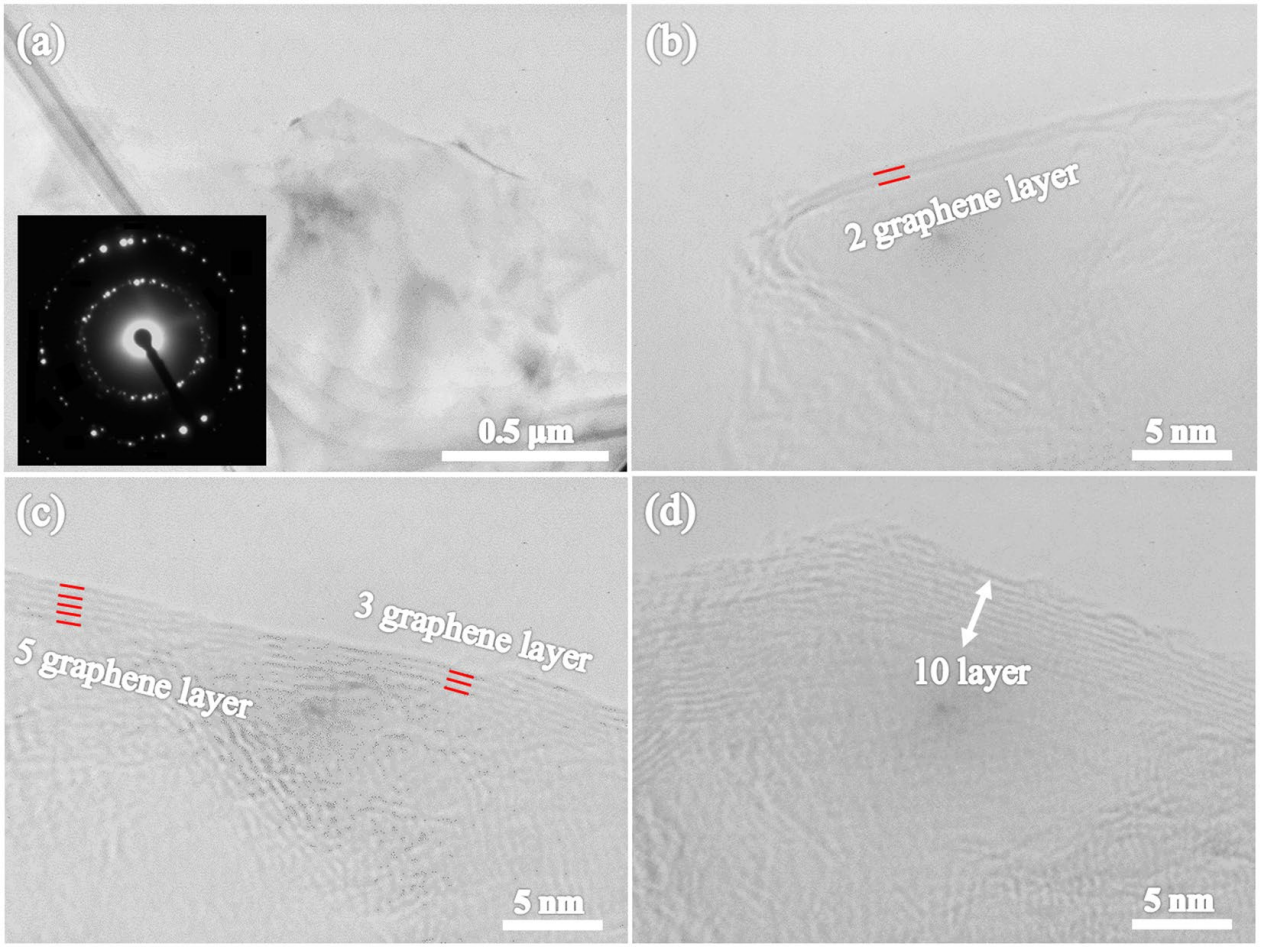
(**a**) Low-magnification TEM image of the carbon nanosheets prepared from 2-pyrrolidone, and the inset is the SAED pattern. High-magnification TEM images showing the edge of carbon nanosheets consisting of multi-layer graphene sheets with (**b**) two, (**c**) five, and (**d**) fifteen layers.

Figure 2(a) shows a low-magnification TEM image of the NCNs prepared from 2-pyrrolidone, and the inset is the SAED pattern. The transparent NCNs were found on the carbon grid, as it was very thin. The SAED pattern suggested that the multiple diffraction spots were caused by the back-folding of edges, intrinsic rotational stacking faults, or overlapping domains of graphene layers^28, 29^. The dispersed bright spots indicated that the NCNs prepared from 2-pyrrolidone mainly consisted of MLG^30^. In order to confirm the graphene layer number, high-magnification TEM images were also obtained. The layer number of graphene can be distinguished from the folded regions of the sheets. Figure 2(b) and (c) reveal the folded edge of the NCNs consisting of graphene sheets with layer numbers of 2–5. In general, the NCNs of this material mainly consisted of MLG as shown in Fig. 2(d).

**Figure 3:**
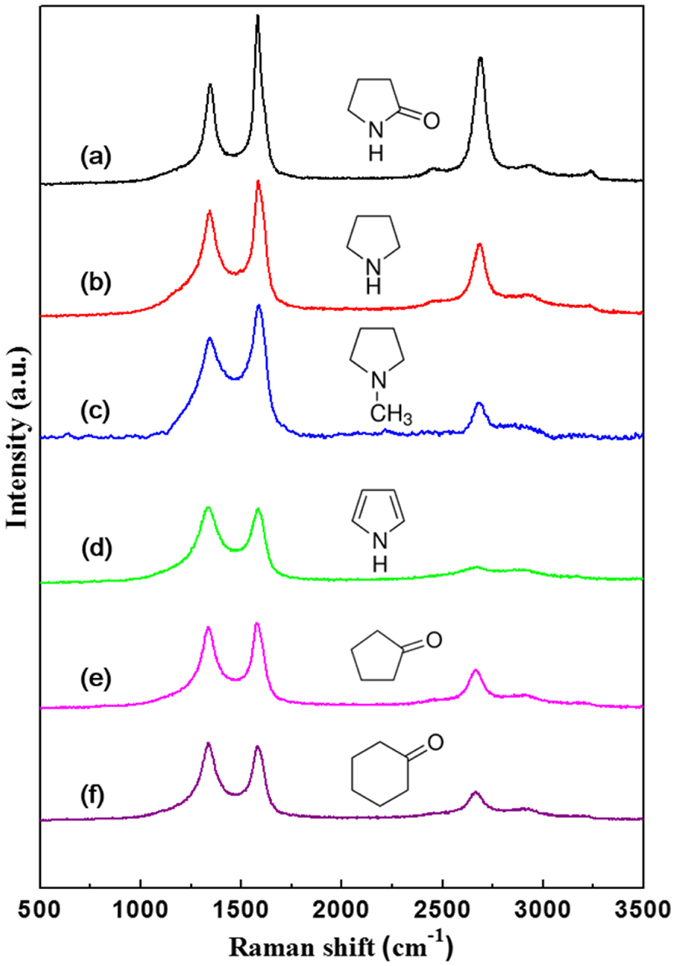
Raman spectra of the carbon nanomaterials obtained from different precursors: (**a**) 2-pyrrolidone, (**b**) pyrrolidine, (**c**) 1-methylpyrrolidine, (**d**) pyrrole, (**e**) cyclopentanone, and (**f**) cyclohexanone.

**Figure 4:**
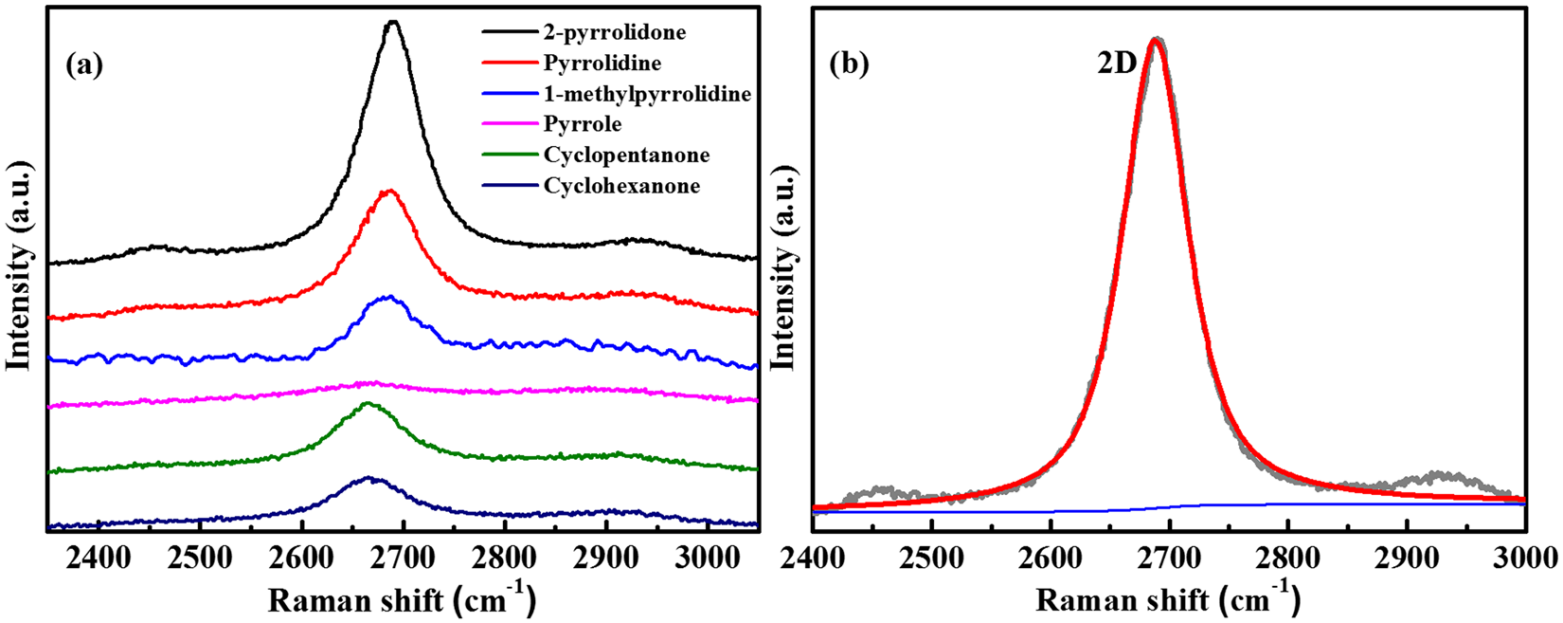
(**a**) 2D-band profile of the carbon nanomaterials prepared from different precursors: 2-pyrrolidone, pyrrolidine, 1-methylpyrrolidine, pyrrole, cyclopentanone, and cyclohexanone. (**b**) 2D-band of the carbon nanosheets prepared from 2-pyrrolidone in a Raman spectrum fitted by a single Lorentzian function.

Typical Raman spectra of the carbon nanomaterials obtained from 2-pyrrolidone, pyrrolidine, 1-methylpyrrolidine, pyrrole, cyclopentanone, and cyclohexanone are presented in Fig. 3. Two fundamental peaks of a D-band and G-band appeared at 1350 cm^−1^ and 1580 cm^−1^, respectively. The G-band corresponds to the E2g mode of sp^2^ bonded carbon atoms in a graphitized structure, whereas the D-band is usually activated by the presence of structural defects and disorder-induced phonon mode in a structure^31–33^. The relative intensity ratio of the D-band to G-band (ID/IG) is measured to analyze the graphitizing degree or defect density of carbon nanomaterials^34^. The ID/IG ratio is usually used to calculate the in-plane crystalline size (La) by the following equation:

1 $${L}_{a}=(2.4\times {10}^{-10}){\lambda }_{l}^{4}{({I}_{D}/{I}_{G})}^{-1},$$

where *λ*_*l*_ is the laser line wavelength in nm^35^. The corresponding *L*_*a*_ values were determined to be 33.3, 24.7, 25.1, 18.7, 20.1, and 18.2 for the carbon nanomaterials prepared from 2-pyrrolidone, pyrrolidine, 1-methylpyrrolidine, pyrrole, cyclopentanone, and cyclohexanone, respectively (Table 1). Previous studies have revealed that the *L*_*a*_ of NCNs prepared from NMP was 32.2 nm, lower than that of nanosheets prepared from 2-pyrrolidone^22^. In this study, the conjugated CH_3_ in the NMP molecular structure was assumed to disrupt the large-area growth of NCNs, whereas both the presence of conjugated oxygen and nitrogen could lead to a more facile formation of nanosheets. A 2D-band in the range of 2600–2800 cm^−1^, which is associated with the second-order of the D-band and corresponds to the presence of a highly ordered carbon lattice, was also observed, as shown in Fig. 3. Figure 4(a) clearly shows that there was a significant difference in the intensity of the 2D-band by comparing the NCNs from 2-pyrrolidone with the carbon nanomaterials from pyrrole. Furthermore, the NCNs from 2-pyrrolidone had the highest *I*_*2D*_ */I*_*G*_ ratio, which indicated graphene quality, whereas the carbon nanomaterials from pyrrole did not have any significant 2D-band signature (Table 1)^33^.

**Table 1:** Summary of surface elemental composition and Raman spectroscopy data of the carbon nanomaterials prepared from different precursors.

Precursor	XPS (at%)			*I*_*D*_/*I*_*G*_	*L*_*a*_ (nm)	*I*_*2D*_ */I*_*G*_	*ω*_*2D*_ (cm^−1^)
	C	O	N				
2-pyrrolidone	93.4	3.6	3.0	0.58	33.3	0.76	66.7
Pyrrolidine	92.5	6.6	0.9	0.78	24.7	0.53	78.0
1-methylpyrrolidine	83.6	8.6	7.8	0.77	25.1	0.25	80.0
Pyrrole	91.2	6.5	2.3	1.03	18.7	—	—
Cyclopentanone	90.7	9.4	0.0	0.96	20.1	0.42	89.2
Cyclohexanone	92.3	7.7	0.0	1.06	18.2	0.36	109.7

The position and intensity of the 2D-band can also be used to identify the number of layers in graphene-related materials^36^. The NCNs prepared from 2-pyrrolidone had a 2D-band, which presented a single symmetrical peak that was a signature of graphene with a full width at half maximum (FWHM, ω2D) of approximately 66.7 cm^−1^ as shown in Fig. 4(b). These results indicate that the NCNs prepared from 2-pyrrolidone may be composed of MLG with turbostratic stacking^37–39^. This turbostratic disorder was also apparent from the results of the X-ray diffraction (XRD), which found an interlayer spacing of 0.346 nm (bulk graphite: 0.335 nm) in the (002) diffraction peak (Fig. S2). The surface area, total pore volume, and pore diameter of the NCNs prepared from 2-pyrrolidone were 321 m^2^ g^−1^, 0.9 cm^3^ g^−1^, and 19 nm, respectively (see Fig. S3 and Table S1).

The surface elemental composition and bonding configuration of carbon nanomaterials were probed by XPS. The XPS survey spectra of the carbon nanomaterials showed predominant narrow peaks of carbon (C 1s) at 284.5 eV, along with nitrogen (N 1s) at 399.5 eV and oxygen (O 1s) at 532.5 eV without any other impurities (Fig. S4). The total N content (at%) of the carbon nanomaterials prepared from 2-pyrrolidone, pyrrolidine, 1-methylpyrrolidine, and pyrrole was 3.0%, 0.9%, 7.8%, and 2.4%, respectively. However, in the case of cyclopentanone and cyclohexanone, nitrogen was not detected (Table 1).

**Figure 5:**
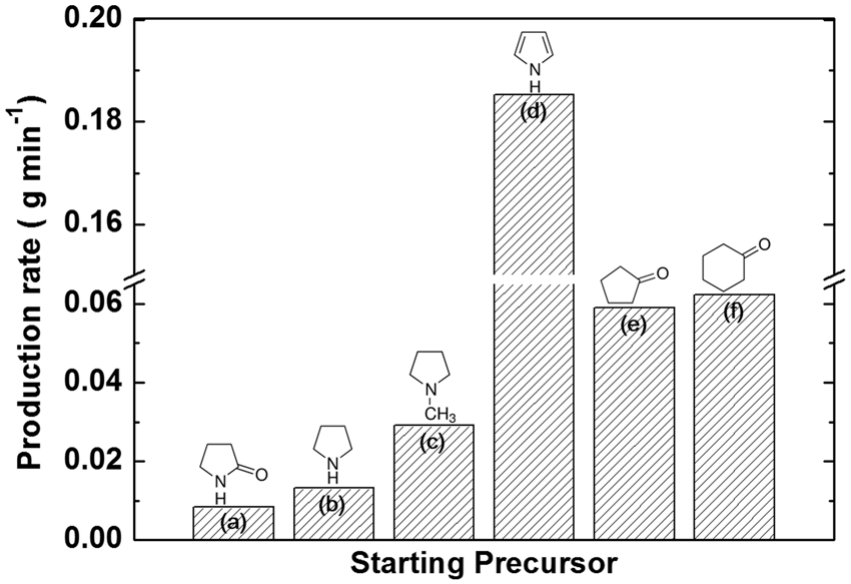
Synthesis rates of carbon nanomaterials obtained from different precursors: (**a**) 2-pyrrolidone, (**b**) pyrrolidine, (**c**) 1-methylpyrrolidine, (**d**) pyrrole, (**e**) cyclopentanone, and (**f**) cyclohexanone.

Figure 5 presents the synthesis rates of carbon nanomaterials obtained from 2-pyrrolidone, pyrrolidine, 1-methylpyrrolidine, pyrrole, cyclopentanone and cyclohexanone (0.0083, 0.0133, 0.0290, 0.1853, 0.0590, and 0.0623 g min^−1^, respectively). Morishita *et al*.^40^ reported a difference in synthesis rates depending on the mechanism of nanocarbon formation via SPP with different starting precursors, as analyzed by gas chromatography-mass spectrometry (GC/MS). They mentioned that the synthesis rate for nanocarbons prepared from ring molecules was substantially greater than that for nanocarbons prepared from linear molecules as a result of different nanocarbon formation routes. This study also found a similar trend: The synthesis rate of carbon nanomaterials from pyrrole was noticeably higher than that of carbon nanomaterials from other starting precursors. The reason for this may have been that the excitation of the π-bonds in saturated ring molecules resulted in the formation of cation radicals, leading to direct polymerization^40, 41^. The lower synthesis rates of carbon nanomaterials from 2-pyrrolidone, pyrrolidine and 1-methylpyrrolidine may be a result of the C–N bonds (293 kJ mol^−1^) in these precursors being easily broken in the solution plasma, leading to the formation of linear molecules^40^. However, a slightly different synthesis route is possible; Panomsuwan *et al*.^42^ used acrylonitrile, which consists of linear molecules, as a starting precursor and they could not synthesize carbon nanosheets. Therefore, a modified carbonization mechanism for carbon nanosheets is required.

The electrical resistivity of the NCNs prepared from 2-pyrrolidone was measured with a device consisting of two probes (model 692A, Metronix Corp.). Carbon powder was placed in a hollow Teflon cylinder with an inner diameter of 5 mm and the electrical resistivity measured while the sample was compressed with a force of 0.6 MPa^43–45^. The resistivity of the NCNs prepared from 2-pyrrolidone was 0.053 Ω·cm, which is of the same order of magnitude as that of carbon nanofibers (CNFs) and carbon nanotubes (CNTs), as shown in Table 2 ^22, 46, 47^. This resistivity also represented an improvement in comparison to NCNs prepared from NMP.

**Table 2:** Electrical resistivity and nitrogen content of NCNs prepared from 2-pyrrolidone, nitrogen-doped carbon nanofibers (CNFs), carbon nanotubes (CNTs), and NCNs prepared from NMP.

Carbon material	Nitrogen	(Ω∙cm) at ambient temperature	Ref.
Nitrogen-doped CNFs	3.1 wt%	0.065	46
Nitrogen-doped CNTs	0.3 at%	~0.040	47
NCNs prepared from NMP	1.3 at%	0.065	22
NCNs prepared from 2-pyrrolidone	3.0 at%	0.053	Present work

**Figure 6:**
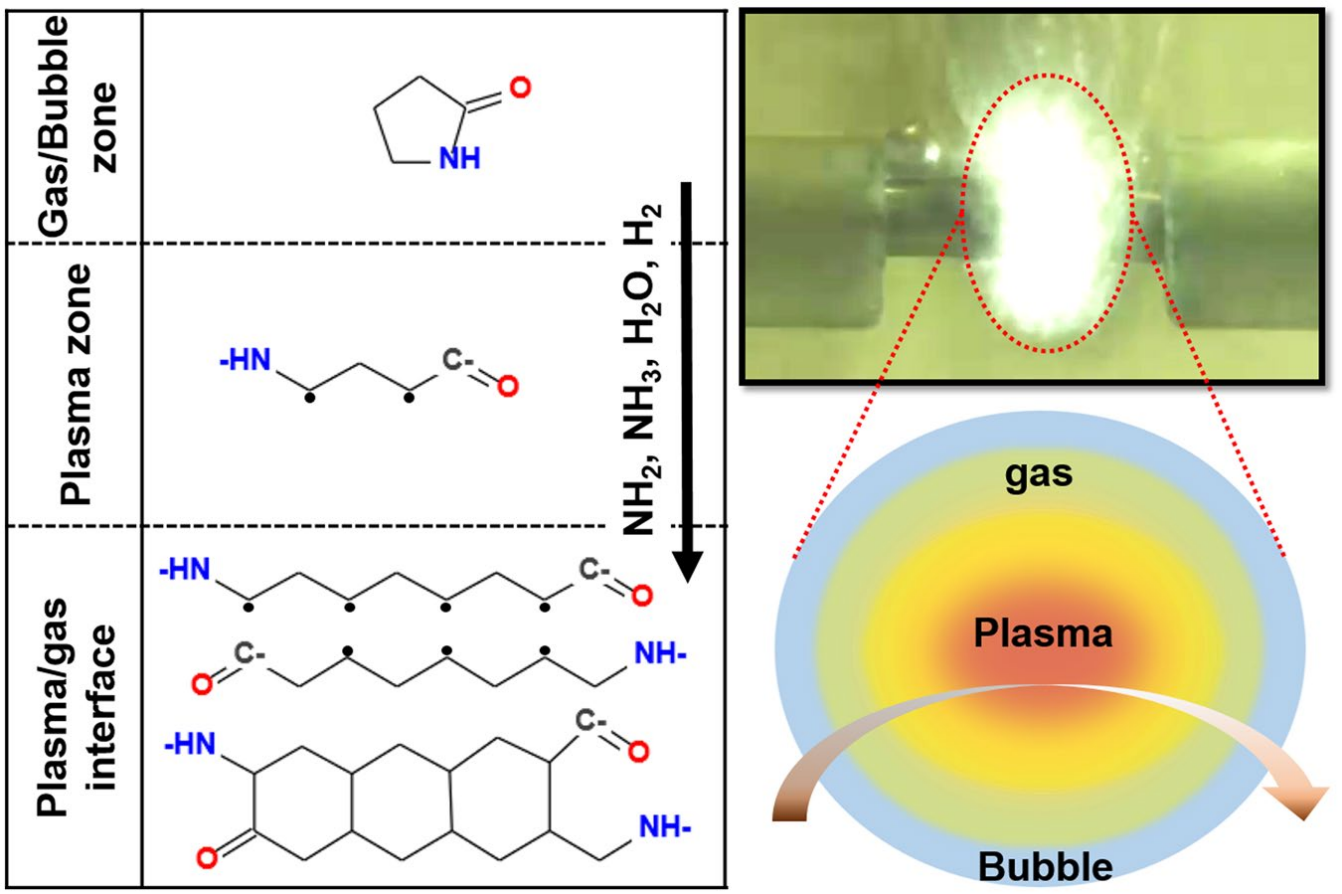
Proposed schematic representation of carbonization process.

The relationships between the results outlined above can be explained as follows. The main reason for the formation of carbon nanosheets was breakage of the C–N bond. The dissociation energy (293 kJ mol^−1^) of the C–N bond is lower than that of the C–C bond (348 kJ mol^−1^). Therefore, the C–N bond in organic precursors such as 2-pyrrolidone was easily broken in solution plasma, as shown in Fig. 6. Further, the precursors were ionized and reacted with each other as highly active radicals in the plasma and plasma/gas interface. During these steps, the carbon residue, formed through the cleavage of the C–N bond, presumably led to the formation of H_2_O, NH_2_ or NH_3_, resulting in long carbon chains. The chains then underwent changes to form a graphite-like structure by intermolecular crosslinking of adjacent chains by dehydrogenation reactions, as shown in Fig. 6. Hence, the SPP with 2-pyrrolidone, pyrrolidine and 1-methylpyrrolidine led to the formation of carbon nanosheets. In addition, when comparing the precursors, the oxygen bond plays a beneficial role in the growth of large-scale and high-quality NCNs. Lee *et al*.^48^ found an inverse relationship between the hydrogen-to-carbon ratio (H/C) and sp^2^ domain size. The reason for the growth of large-scale and high-quality NCNs may be that the amount of hydrogen was further reduced by the elimination of the H_2_O that was generated in the reaction between hydrogen and oxygen, as shown in Table S2^25, 48, 49^. This is why the NCNs prepared from pyrrolidine and 1-methylpyrrolidine, which have greater H/C ratios, produced randomly aggregated, dense, and thick nanosheets of irregular shapes.

On the other hand, the SPP with cyclopentanone, cyclohexanone and pyrrole did not lead to the formation of carbon nanosheets owing to the greater dissociation energy of the C–C bond than of the C–N bond, resulting in a reaction without bond breakage. Kroto *et al*.^50^ demonstrated that the presence of pentagon carbon rings in the nucleation process results in the growth of spiral shells, which can be considered a mechanism for carbon anion formation. In the same vein, Kang *et al*.^51^ reported that the mechanism behind the formation of carbon spheres prepared from benzene using SPP was confirmed by the identification of evidence of compounds which can be used as precursors for the growth of quasi-icosahedral shells^52–54^. Hence, the formation mechanism of carbon nanoballs from cyclopentanone, cyclohexanone, and pyrrole, which are ring molecules like benzene^40^, was also proposed in a similar manner.

## Discussion

In this study, various precursors were used in the SPP to verify the effect of elementary composition and structure of NMP in the formation of NCNs. 2-pyrrolidone in the various precursors was demonstrated to facilitate the formation of highly ordered NCNs. The main reason for the formation of carbon nanosheets may have been that C–N bond was broken by SPP. And the oxygen bond played a beneficial role in the large-scale and high-quality growth of NCNs. The results of this study would uncover new parameter fields for the growth of heteroatom-CNs using this synthesis system. In addition, this study is expected to contribute toward research in improving the large-area growth and quality of 2D nanostructures, such as heteroatom-CNs or graphene, for various applications in other synthesis methods.

## Methods

### Chemicals

The chemicals, 2-pyrrolidone (C_4_H_7_NO, 99.0%), pyrrolidine (C_4_H_9_N, 99.0%), 1-methylpyrrolidine (C_5_H_11_N, 99.0%), pyrrole (C_4_H_5_N, 99.0%), cyclopentanone (C_5_H_8_O, 99.0%), and cyclohexanone (C_6_H_10_O, 99.0%), were purchased from a commercial supplier, Tokyo Chemical Industry (TCI, Japan).

### The SPP and synthesis of carbon nanomaterials

**Figure 7:**
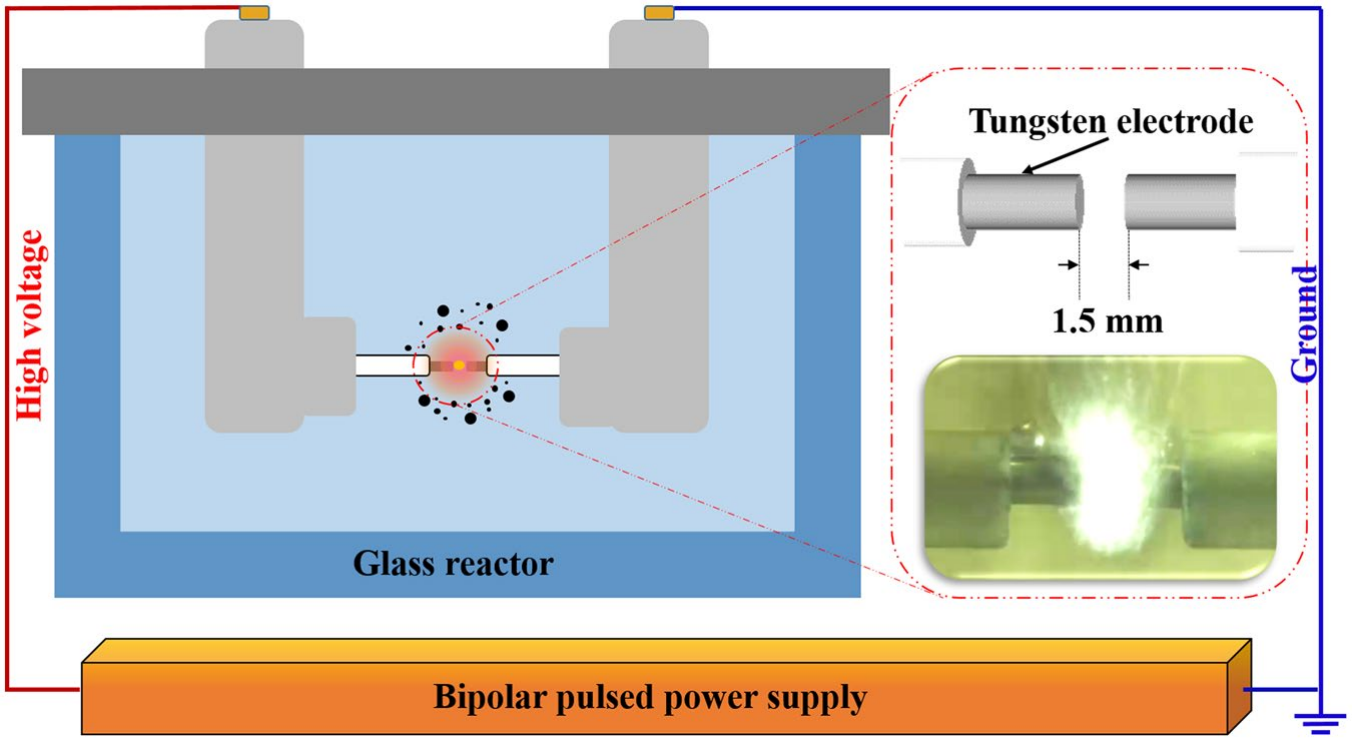
Schematic diagram of the experimental setup of the solution plasma process.

**Figure 8:**
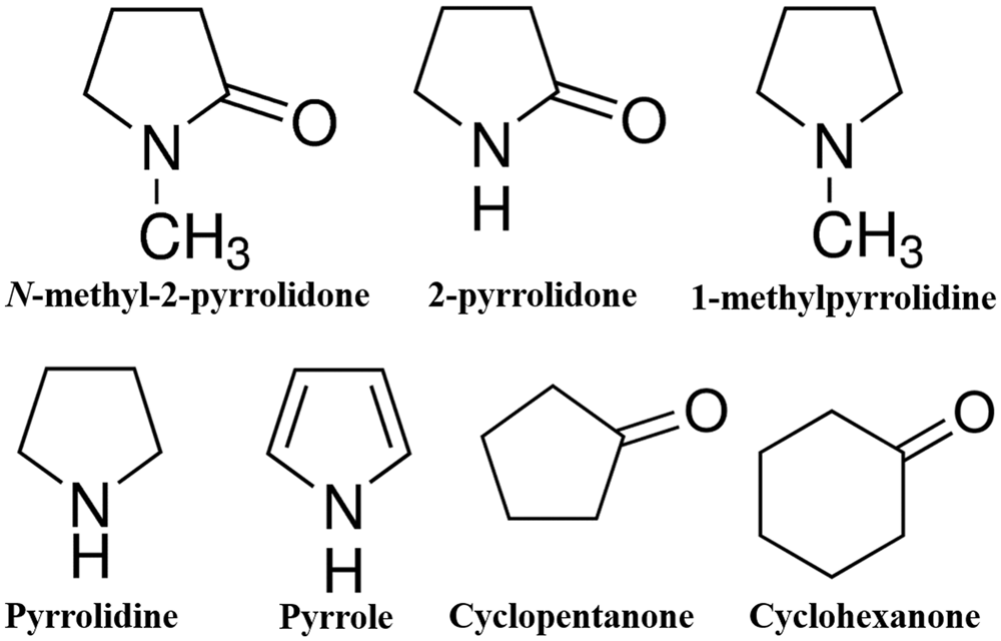
Precursors used in the solution plasma process (SPP): *N*-methyl-2-pyrrolidone, 2-pyrrolidone, 1-methylpyrrolidine, pyrrolidine, pyrrole, cyclopentanone, and cyclohexanone.

Figure 7 shows the experimental setup of the SPP used to synthesize carbon nanomaterials. The setup consisted of two tungsten electrodes (diameter: 1 mm) that were located at a distance of 1.5 mm between each other in a glass reactor. The glass reactor was filled with 200 mL volume of various aqueous precursors as carbon or nitrogen source. A bipolar pulsed power supply (Kurita, Japan) was used to generate the plasma in the corresponding solution at atmospheric pressure and room temperature conditions. The repetition pulse width, frequency, and voltage applied to the electrodes for 5 min were fixed at 1.0 μs, 200 kHz, and 2.0 kV, respectively. After the discharge, the black carbon particles were separated by vacuum filtration through a polytetrafluoroethylene (PTFE) membrane filter (pore size of 100 nm). The as-synthesized carbon nanomaterials were then dried in an oven at 200 °C for 1 h. Figure 8 depicts the various precursors, which have a similar molecule structure to NMP, as carbon or nitrogen source for the synthesis of carbon nanomaterials in this study. As mentioned above, the precursors were used to determine whether the nature of a precursor determines the shape of obtained carbon nanomaterials. 2-pyrrolidone is a precursor without a CH_3_ bond. 1-methylpyrrolidine is a precursor without an oxygen bond. Pyrrolidine is a precursor without both CH_3_ and oxygen bonds. Pyrrole is a heterocyclic aromatic organic compound. Cyclopentanone and cyclohexanone are carbocyclic compounds that have a 5- and 6-membered ring with a double bonded oxygen, respectively.

### Characterization

TEM images and selected area electron diffraction (SAED) patterns were obtained from a JEOL-2500SE microscope using an accelerating voltage of 200 kV. Raman spectra were collected by a JASCO NRS-100 spectrometer at an excitation wavelength of 532.5 nm. XPS measurements were performed with a ULVAC PHI 5000 VersaProbe II using a Mg Kα X- ray source.

## Electronic supplementary material

supplementary information
