# Supplementary material for: The solution plasma process for heteroatom-carbon nanosheets: the role of precursors
Source: Sci Rep. 2017 Jun 19;7:3825. doi: 10.1038/s41598-017-04190-x (PMC5476662; doi:10.1038/s41598-017-04190-x)
Supplement: Supplementary file 1 — supplementary information [file 41598_2017_4190_MOESM1_ESM.doc]

***Supplementary information***

The solution plasma process for heteroatom-carbon nanosheets: the role of precursors

Koangyong Hyun^1*^, Nagahiro Saito^2^

^1^Faculty of Engineering, Shinshu University, Nagano 380-8553, Japan.

^2^Graduate School of Engineering, Nagoya University, Nagoya 464-8603, Japan.

^*^kyhyun@shinshu-u.ac.jp

**Characterizations**

Field-emission scanning electron microscopy (FE-SEM) images were taken on JEOL JSM7500FA at an accelerating voltage of 15kV. The X-ray diffraction (XRD) patterns were recorded on Rigaku Smartlab with Cu K_α_ radiation (λ = 0.154 nm). Brunauer-Emmett-Teller (BET, Belsorp-mini II; Belsorp) were conducted to determine the specific surface areas, pore volume, and pore sizes. Elemental analysis of C, H and N was performed using an elemental analyzer (Perkin Elmer 2400 Series).

**Results**

**
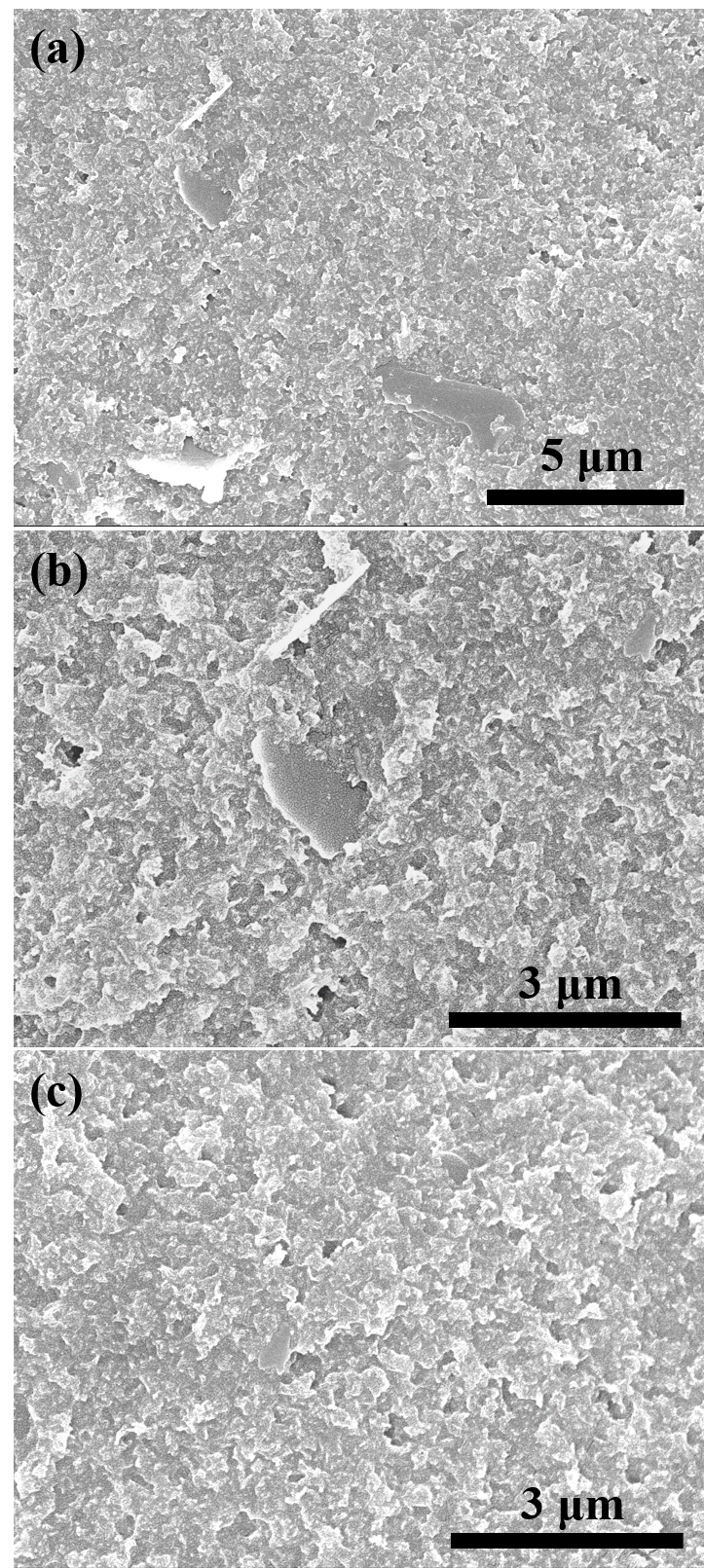
**

**Figure S1.** FE-SEM images of NCNs prepared from 2-pyrrolidone.


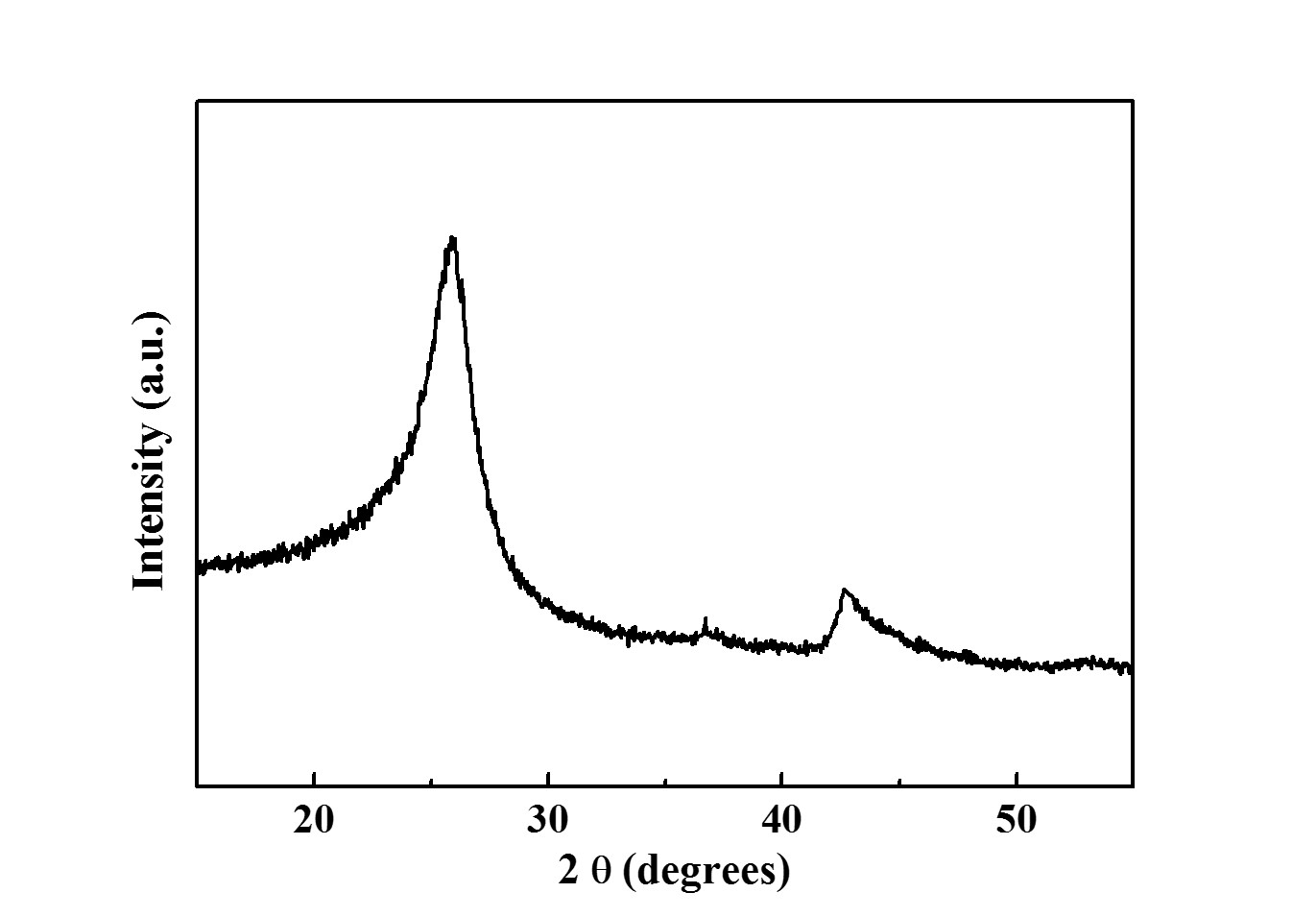


**Figure S2.** X-ray diffraction (XRD) for NCNs prepared from 2-pyrrolidone.

*
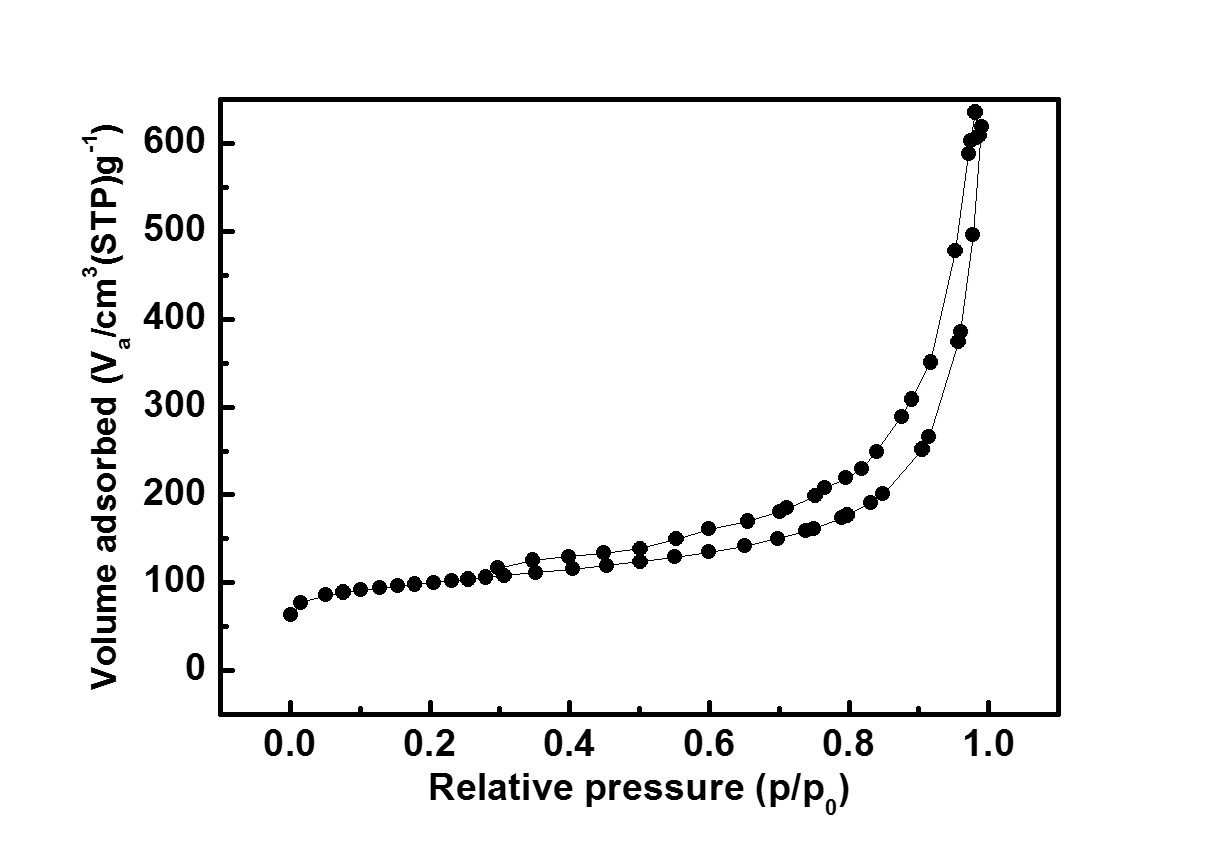
*

**Figure S3.** Nitrogen adsorption-desorption isotherms for NCNs prepared from 2-pyrrolidone.

| Material | Surface area  (m^2^g^–1^) | Pore volume  (cm^3^g^–1^) | Mean pore diameter  (nm) |
| --- | --- | --- | --- |
| NCNs prepared from 2-pyrrolidone | 321 | 0.9 | 19 |

**Table S1.** Structural parameters of NCNs prepared from 2-pyrrolidone.

| Starting precursor | Elementary Analysis (wt%) | | | H/C (mol/mol)^a^ of starting precursor | H/C (mol/mol)^b^ of as-prepared carbon | Reduction rate (%)^c^ of H/C |
| --- | --- | --- | --- | --- | --- | --- |
|  | C | H | N |  |  |  |
| 2-pyrrolidone | 92.71 | 0.68 | 2.39 | 1.75 | 0.088 | 95.0 |
| Pyrrolidine | 90.96 | 1.23 | 1.84 | 2.25 | 0.162 | 92.8 |
| 1-methyl-pyrrolidine | 78.94 | 3.62 | 10.43 | 2.20 | 0.550 | 75.0 |
| c = (a – b) / a ×100 | | | | | | |

**Table S2.** Results of an elementary analysis (EA) of as-prepared carbon powders produced from 2-pyrrolidone, pyrrolidine and 1-methylpyrrolidine and the reduction in the hydrogen-to-carbon ratio (H/C) from the starting precursor to the as-prepared carbon powder.


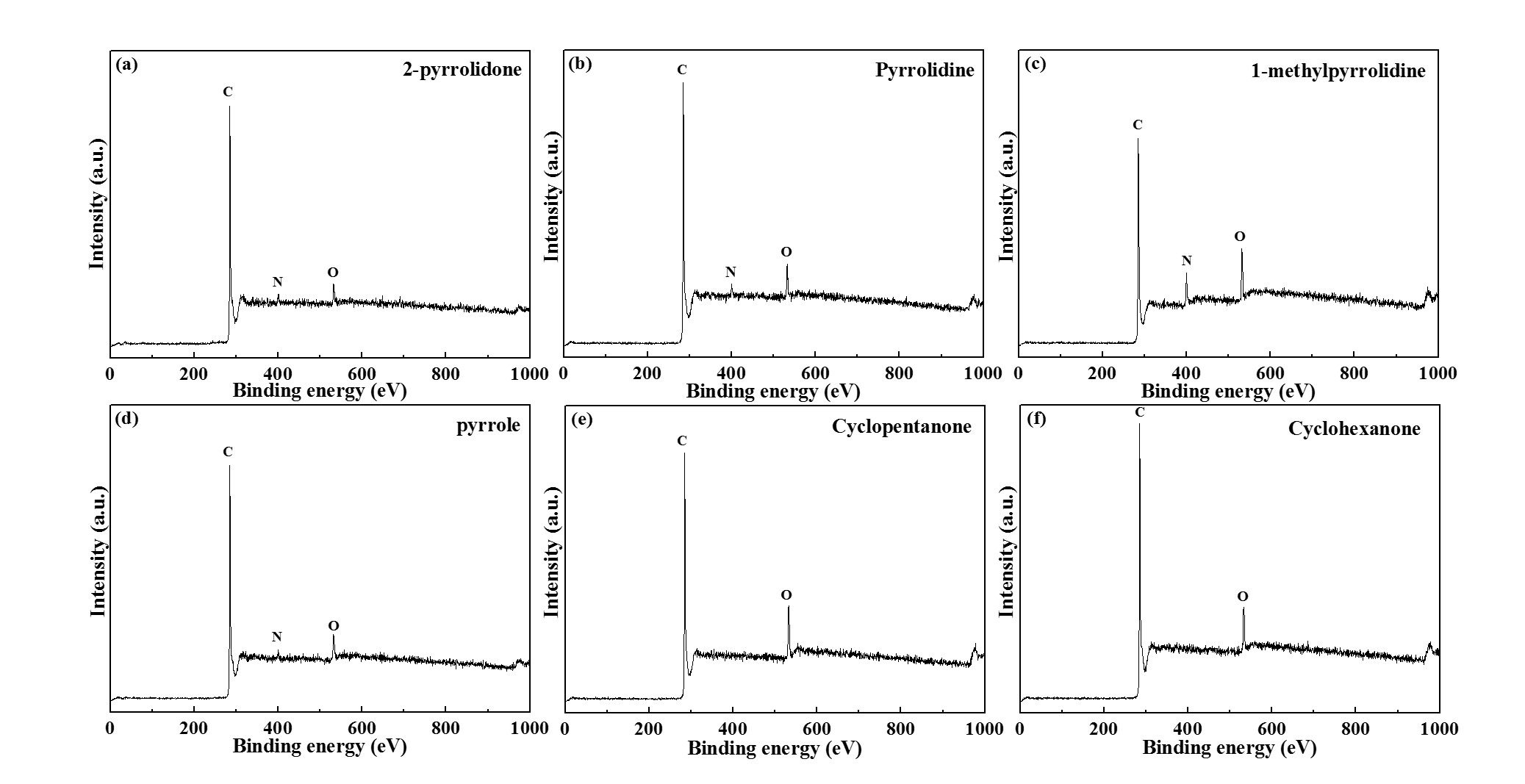


**Figure S4.** Survey X-ray photoelectron spectroscopy (XPS) spectra of the carbon nanomaterials prepared from different precursors: (a) 2-pyrrolidone, (b) pyrrolidine, (c) 1-methylpyrrolidine, (d) pyrrole, (e) cyclopentanone, and (f) cyclohexanone.


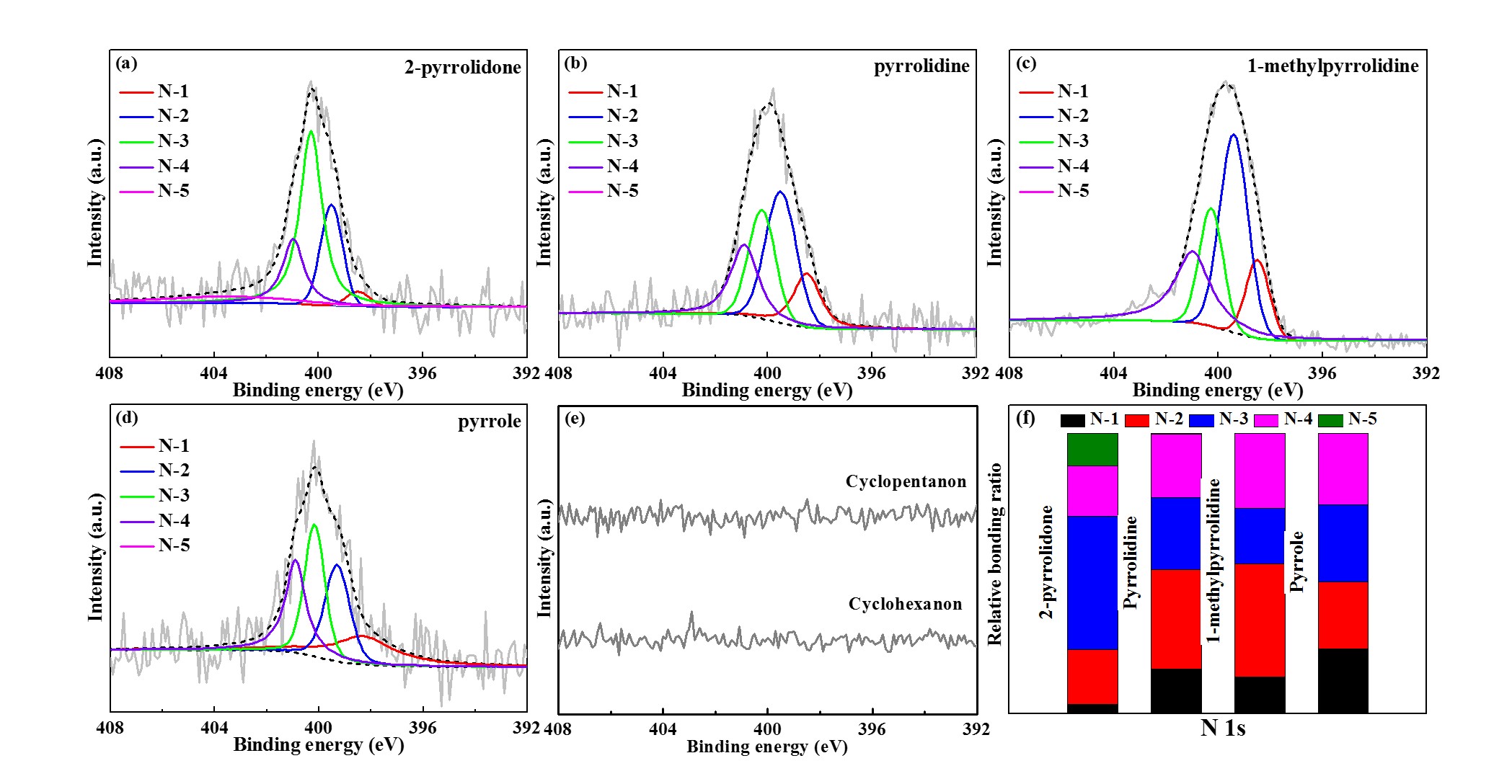


**Figure S5.** High-resolution N 1s XPS spectra of the carbon nanomaterials prepared from different precursors: (a) 2-pyrrolidone, (b) pyrrolidine, (c) 1-methylpyrrolidine, (d) pyrrole, (e) cyclopentanone, and cyclohexanone. (f) Relative bonding ratio of each N coordination species.


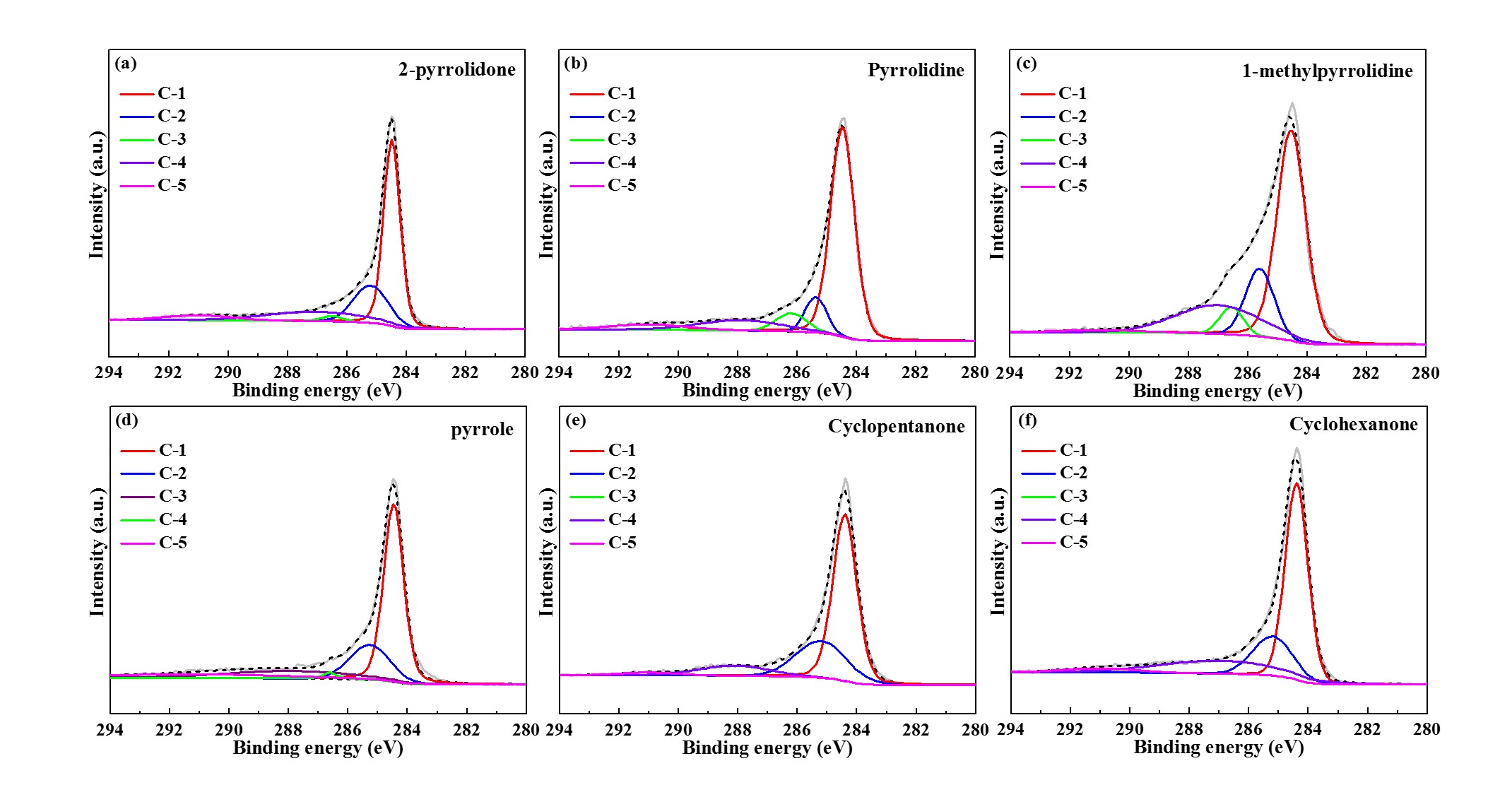


**Figure S6.** High-resolution C 1s XPS spectra of the carbon nanomaterials prepared from different precursors: (a) 2-pyrrolidone, (b) pyrrolidine, (c) 1-methylpyrrolidine, (d) pyrrole, (e) cyclopentanone, and (f) cyclohexanone.


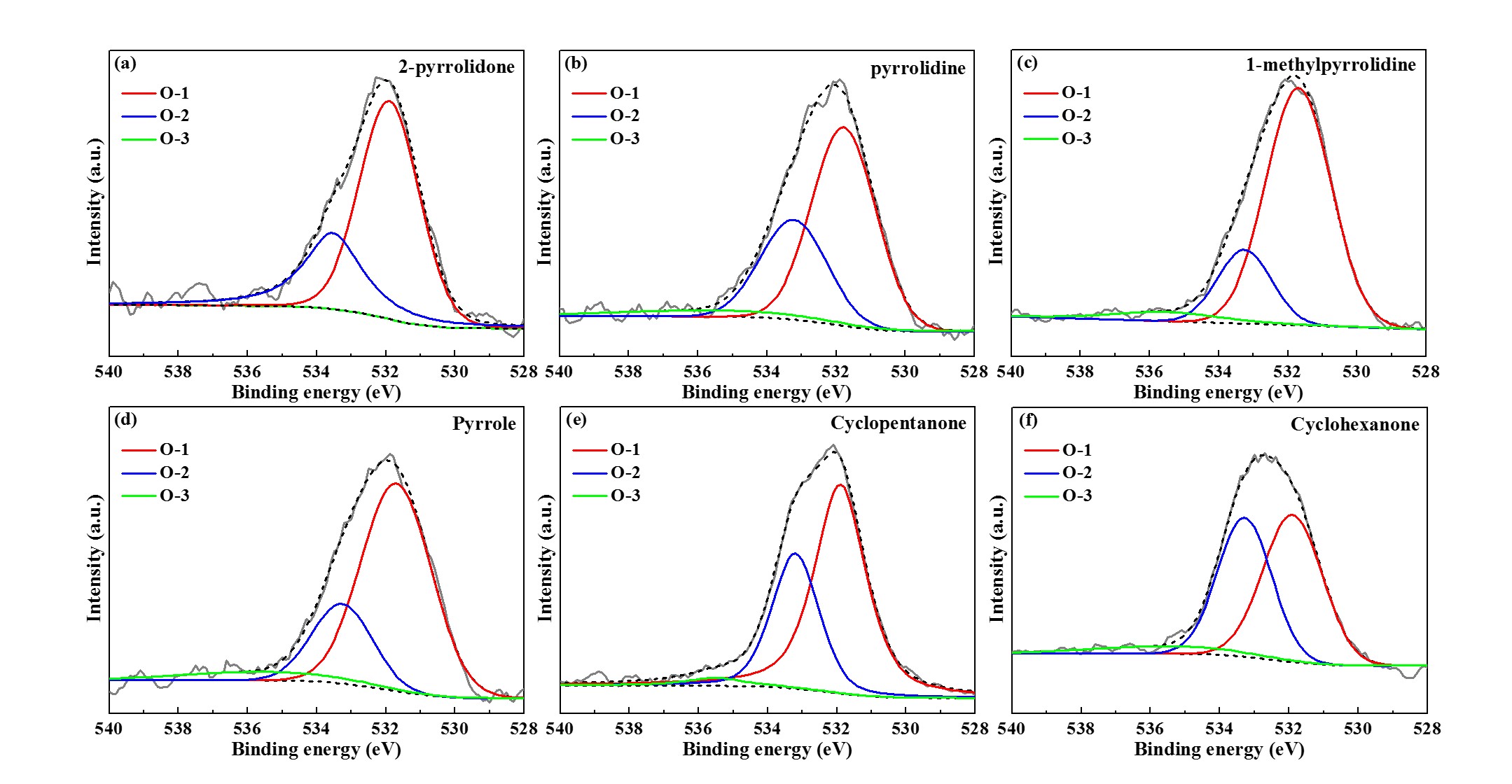


**Figure S7.** High-resolution O 1s XPS spectra of the carbon nanomaterials prepared from different precursors: (a) 2-pyrrolidone, (b) pyrrolidine, (c) 1-methylpyrrolidine, (d) pyrrole, (e) cyclopentanone, and (f) cyclohexanone.

The high-resolution N 1s XPS spectra in Fig. S5 shows the presence of the several types of N coordination such as pyridinic-N (N-1, 398.4 ± 0.1eV), nitrile-N (N-2, 399.4 ± 0.1 eV), pyrrolic-N (N-3, 400.1 ± 0.2 eV), graphitic-N (N-4, 401.0 ± 0.1 eV), and oxidized-N (N-5, 403.7 ± 0.2 eV)^1–6^. The relative chemical bonding ratio of N species was calculated to evaluate the corresponding type and level, as shown in Fig. S5(f). Remarkably, the N-2 configuration was the dominant site in the carbon nanomaterials prepared from pyrrolidine and 1-methylpyrrolidine as shown in Fig. S5(b) and S5(c), respectively. On the other hand, in the case of 2-pyrrolidone, the N-3 configuration was predominant, and it had the N-5 configuration that was not found in other precursors. The reason for this difference may be that the amount of hydrogen was further reduced by the elimination of the H_2_O that was generated in the reaction between hydrogen and oxygen, as shown in Table S2. Most of the carbon nanomaterials had a high-resolution C 1s peak at 284.5 ± 0.1 eV (C-1) corresponding to the sp^2^ graphite structural form, along with other small peaks at 285.4 ± 0.2 eV (C-2), 286.4 ± 0.2 eV (C-3), 288.0 ± 0.1 eV (C-4), and 290.7–291.9 eV (C-5) corresponding to the presence of C sp^3^ bond, C–O/C=N, C–N/C=O, and C=O bonds of different types (carbonyl, aldehyde, *etc.*), respectively^1,7–9^. In particular, C-3 was only observed in the carbon nanomaterials prepared from precursors with a nitrogen atom in the molecular structure (Fig. S6). The high-resolution O 1s peak shown in Fig. S7 was composed of several types of O coordination such as O-1 (C=O/O–C, 531.8 ± 0.1 eV), O-2 (C–O–C/COOH/C–OH, 532.1 eV), and O-3 (adsorbed oxygen or water, 535.5 eV)^1,7^.

**References**

1. Panomsuwan, G., Saito, N., Ishizaki, T. Nitrogen-doped carbon nanoparticles derived from acrylonitrile plasma for electrochemical oxygen reduction. *Phys. Chem. Chem. Phys.* **17**, 6227–6232 (2015).
2. Ding, W. *et al.* Space-confinement-induced synthesis of pyridinic- and pyrrolic-nitrogen-doped graphene for the catalysis of oxygen reduction. *Angew. Chem., Int. Ed.* **52,** 11755–11759 (2013).
3. Pels, J. R., Kapteijn, F., Moulijn, J. A., Zhu, Q., Thomas, K. M. Evolution of nitrogen functionalities in carbonaceous materials during pyrolysis. *Carbon* **33**, 1641–1653 (1995).
4. Wei, D. *et al.* Synthesis of N-doped graphene by chemical vapor deposition and its electrical properties. *Nano Lett.* **9**, 1752–1758 (2009).
5. Zhang, C. *et al.* Synthesis of nitrogen-doped graphene using embedded carbon and nitrogen sources. *Adv. Mater.* **23,** 1020–1024 (2011).
6. Lu, Y. F. *et al.* Nitrogen-doped graphene sheets grown by chemical vapor deposition: synthesis and influence of nitrogen impurities on carrier transport. *ACS Nano* **7**, 6522–6532 (2013).
7. Datsynk, V. *et al.* Chemical oxidation of multiwalled carbon nanotubes. *Carbon* **46**, 833–840 (2008).
8. Beamson, G., Grigg, D. High resolution XPS of organic polymers. The scienta ESCA A300 database, Chichester: Wiley 1992.
9. Velázquez-Palenzuela, A. *et al.* Carbon-supported Fe–N_x_ catalysts synthesized by pyrolysis of the Fe(II)–2,3,5,6-tetra(2-pyridyl)pyrazine complex: structure, electrochemical properties, and oxygen reduction reaction activity. *J. Phys. Chem. C* **115,** 12929–12940 (2011).
